# Investigation and control of a measles outbreak at the Hong Kong Special Administrative Region International Airport, 2019

**DOI:** 10.5365/wpsar.2019.10.2.007

**Published:** 2020-06-29

**Authors:** Wong Chi Hong, Chuang Shuk Kwan, Lam Wing Hang, Lam Ho Yeung, Lam Tsz Sum, Ho Lei Ming Raymond, Leung Yiu Hong, Lam Chau Kuen Yonnie

**Affiliations:** a Centre for Health Protection, Department of Health, Hong Kong SAR (China).; b Field Epidemiology Training Programme, Hong Kong SAR (China).

## Abstract

**Introduction:**

Hong Kong Special Administrative Region SAR (China) achieved measles elimination status in 2016, and the incidence of measles infection had been low over the past few years. However, the Centre for Health Protection (CHP) at the Department of Health was notified on 22 March 2019 of an outbreak of three cases of measles infection among workers at the Hong Kong Special Administrative Region International Airport (HKIA).

**Methods:**

We reviewed notifications of measles received by CHP from 1 January to 17 May 2019. We defined a confirmed case of measles as having laboratory evidence of measles infection. All confirmed cases among airport workers or those with epidemiological information suggesting they had been infected by contact with airport workers were included in the review. We described the epidemiological features and reviewed the control measures against the outbreak.

**Results:**

We identified 33 cases, 29 of which were among airport workers. They comprised 22 men and 11 women, aged 20–49 years (median 25 years). The majority of people with confirmed measles presented with fever and rash. All required hospitalization. None developed complications. Control measures, including enhanced environmental hygiene and improved ventilation at HKIA and vaccinations for the airport community, were implemented. Vaccinations were provided to 8501 eligible airport workers, and the outbreak was declared over on 17 May 2019.

**Discussion:**

Early recognition of the outbreak and prompt control measures, especially targeted vaccination of the exposed population, effectively controlled the outbreak in just two weeks.

Hong Kong Special Administrative Region SAR (China) achieved measles elimination in 2016. The annual number of measles cases had remained at a very low level since then, with nine, four and 15 cases recorded in 2016, 2017 and 2018, respectively. In 2019, amid worldwide increases in measles incidence, especially in the Philippines, the Centre for Health Protection (CHP) of the Department of Health of Hong Kong Special Administrative Region SAR (China) also recorded an upsurge of measles cases (73 cases as of 17 May 2019), including a major outbreak at the Hong Kong Special Administrative Region International Airport (HKIA).

HKIA occupies 1255 hectares on Lantau Island. It is one of the world’s largest and busiest airports, connecting 120 airlines to over 220 destinations worldwide and handling about 75 million passengers in 2018. It has more than 73 000 workers. CHP was notified on 22 March 2019 of an outbreak of three cases among HKIA workers, and an epidemiological investigation was initiated.

## Methods

### Case definition

For this investigation, we defined a laboratory-confirmed case of measles as a person having any of the following: (1) a positive serological test for measles virus IgM antibody; (2) a fourfold or greater increase in the measles antibody (IgG) titre; (3) the isolation of measles virus from a clinical specimen; or (4) a positive reverse transcription-polymerase chain reaction (RT–PCR) for measles virus in a clinical specimen, with any of the four occurring between 11 February and 17 May 2019.

Typical measles was defined as a patient with laboratory-confirmed measles who presented with fever, rash and at least one of the three “C”s (cough, coryza or conjunctivitis). Patients with laboratory-confirmed measles who did not have signs or symptoms satisfying the definition of typical measles were classified as having modified measles.

### Study period and case selection

The earliest recorded confirmed measles patient among the HKIA workers had an onset of rash on 4 March 2019. In an effort to identify any other epidemiologically linked measles cases, we reviewed all measles cases notified to CHP from 1 January to 17 May 2019. All cases among the HKIA workers were included in the HKIA outbreak investigation. Patients with epidemiological information suggesting that they were infected or contracted the disease from an airport worker were considered to be epidemiologically linked to the HKIA outbreak.

We conducted an epidemiological investigation for every measles case. We reviewed the clinical records and interviewed patients for demographic information and their clinical course, travel history, exposure and contact history. We investigated the local movements of all patients during the incubation and communicable periods, attempting to postulate the transmission chain of the outbreak. We also reviewed CHP records for the timing and type of control measures implemented during the outbreak.

### Ethics statement

Ethics approval was not required as this was an emergency response case.

## Results

### The cases

**Figure 1:**
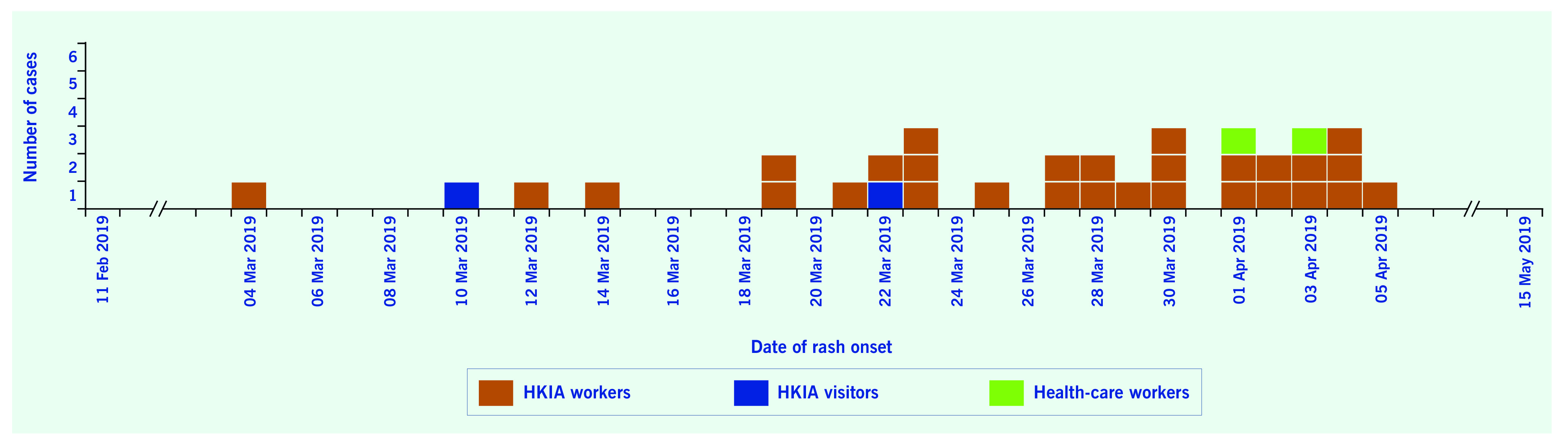
**Epidemic curve of measles cases linked to the HKIA outbreak, 2019 (by date of rash onset)**

We identified 29 cases among airport workers in the HKIA outbreak and four cases epidemiologically linked to the outbreak (one airport visitor, one traveller and two health-care workers with nosocomial exposure to an airport case). These 33 cases comprised 22 men and 11 women, aged 20–49 years (median 25 years). Two thirds (22/33) of the patients were aged 20–29 years. The first patient had an onset of rash on 4 March 2019, and the last patient had an onset of rash on 5 April 2019 (**Fig. 1**). Most had rash (33, 100%) and fever (31, 93.9%). Their clinical courses were mild and none developed complications. All were isolated in a hospital until the end of the communicable period (four days after the onset of rash). All respiratory specimens from the 33 patients tested positive for measles virus by polymerase chain reaction (PCR) and belonged to genotype B3.

[**insert** Figure 1]

The measles vaccination history of the 33 patients showed that 12 (36%) had a documented record of at least two doses of measles-containing vaccine, 19 (58%) had no officially documented history of vaccination, and two (6%) were unvaccinated. Among the 33 cases, 23 (70%) were born in Hong Kong Special Administrative Region SAR (China) and 10 (30%) were not born locally.

Fifteen cases (45%) were classified as typical measles, and 18 (55%) were modified measles. Nine (50%) patients among the 18 modified measles cases and three (20%) among the 15 typical measles cases had received two or more doses of measles-containing vaccine.

**Figure 2:**
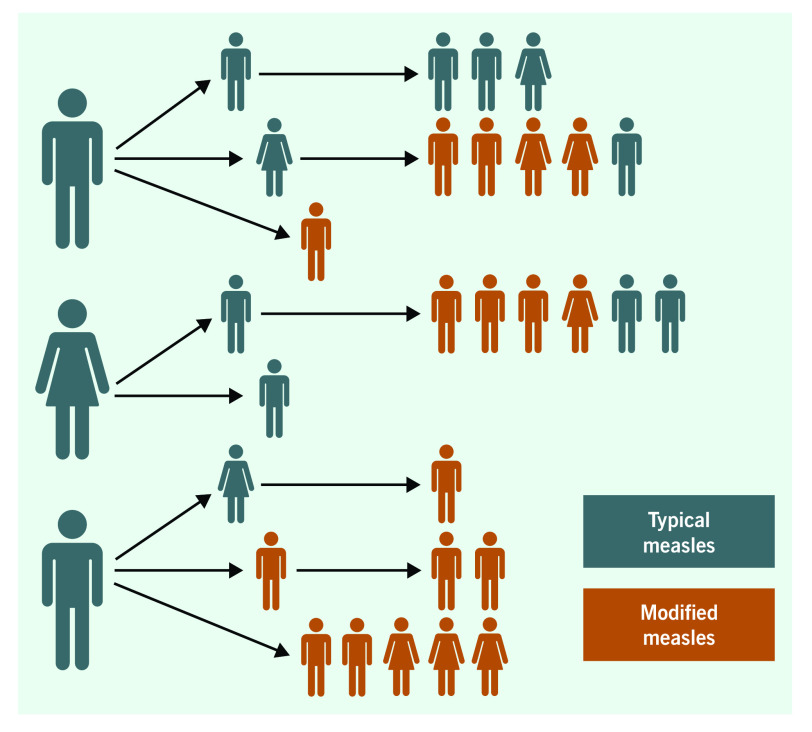
**Transmission chain of HKIA outbreak (n = 32)**

Most (27/29) of the affected airport workers did not know each other and could not recall any direct contact with other affected individuals. We identified at least three subclusters of this outbreak, with separate sources of infection affecting 32 of the 33 cases. Each of the suspected sources was responsible for two generations of infection and affected one to seven people in each generation (**Fig. 2**). For one case, the source could not be determined.

[**insert** Figure 2]

### Outbreak response

The measles outbreak at HKIA was confirmed on 22 March 2019 when three measles cases were notified to CHP and an initial epidemiological investigation revealed that the affected individuals were all airport workers who likely contracted measles at work. An outbreak response team was formed on the same day to carry out in-depth epidemiological investigations and formulate targeted control measures. An onsite investigation was conducted with experts in microbiology and field epidemiology. HKIA management was advised to improve ventilation by increasing the intake of fresh air and increasing the number of alcohol-based hand-rub dispensers in the airport. Press releases alerted the public to the measles outbreak and provided information about prevention and control measures.

Immediately after the outbreak was identified, measles-mumps-rubella (MMR) vaccinations were offered to airport workers without presumptive measles immunity, for example, those without a history of vaccination, as an outbreak control measure. Vaccination stations were set up at the airport, and medical teams, including doctors and nurses, were deployed to conduct onsite vaccinations. Between 22 March and 17 May 2019, MMR vaccinations were provided to 8501 airport workers.

The outbreak was declared over on 17 May after two incubation periods (42 days) passed since 5 April 2019, the date when the last case visited HKIA.

## Discussion

This was the first major outbreak recorded in Hong Kong Special Administrative Region SAR (China) since the certification of the elimination of measles in 2016. Outbreaks among workers in airports have been reported previously elsewhere, for example, in Kansai, Japan, in 2016, affecting 34 individuals (including 32 airport staff members and two health-care workers) and Taoyuan International Airport in China, Taiwan (China) in 2018. (*1*, *2*) Heavy traffic flows, crowded environments that include international travellers and the recent upsurge in measles cases worldwide put airport workers at higher risk than the general population of having contact with travellers infected with measles. The airport’s recirculating ventilation design and crowded environments in certain places, such as changing rooms, might have contributed to the transmission of measles among the HKIA workers who shared the same air space but might not have close interaction with one another. Measles virus can live up to two hours in airspace where an infected person has coughed or sneezed. (*3*) Susceptible individuals may become infected by breathing contaminated air and/or touching contaminated environments.

More than half of the cases (55%) in this outbreak were classified as modified measles, which is considered to have lower transmission potential. (*4*) This is consistent with our observation that most of the patients who gave rise to secondary cases presented with clinically typical measles.

Primary vaccine failure occurs in some recipients of measles-containing vaccine, with about 5% of people who received two doses of measles vaccines not developing immunity after vaccination. (*5*) One study has suggested that in the post-elimination era, when there is lack of boosting of immunity from exposure of wild-type measles, the duration of immunity among vaccinated individuals may not last. (*6*) Moreover, recent studies also supported the presence of secondary vaccine failure, in which waning immunity in adults who received two doses of measles-containing vaccine was observed. (*7*, *8*) Among the 33 affected individuals, two thirds (22/33) were 20–29 years old, and more than half (12/22) of them had a documented history of having previously received two or more doses of measles-containing vaccine. Further analysis of the IgM and IgG results from blood specimens taken within 72 hours of rash onset could provide more information on the proportion of cases with potential secondary vaccine failure.

This outbreak lasted for a month, from 4 March, when the first patient had an onset of rash, to 5 April, when the last patient had an onset of rash, and the outbreak was halted after two generations of transmission. We believe that early recognition of the outbreak and prompt implementation of control measures, especially the aggressive vaccination campaign targeted at airport staff, effectively prevented further spread of the disease and swiftly controlled the outbreak in about two weeks – from the identification of the outbreak on 22 March to 5 April, when the last affected individual visited HKIA.

One limitation of this report is the fact that our analysis of the transmission was retrospective, based on self-reported local movement history provided by the patients. Such reporting is subject to recall error and might not reflect the actual transmission chain. Because of the mild clinical course of the cases, other undiagnosed measles cases likely existed but were not detected, which may underestimate the actual outbreak size.

It is possible that other people may have been infected through this outbreak and travelled outside of Hong Kong Special Administrative Region SAR (China) and, therefore, would not have been included in this study. Cross-border communication of measles outbreaks involving other airports might have provided data to plug the loophole and better reflect the actual outbreak situation.

## Conclusions

Measles remains a public health threat, even in areas where measles has been eliminated. We demonstrated that early recognition of an outbreak and prompt control measures, especially vaccination for a potentially exposed population, can quickly control measles outbreaks.
